# New strategy for silicon supply through fertigation in sugarcane integrating the pre-sprouted seedling phase and field cultivation

**DOI:** 10.1038/s41598-022-27323-3

**Published:** 2023-01-21

**Authors:** José Lucas Farias da Silva, Renato de Mello Prado, Thayane Leonel Alves, Luis Felipe Lata-Tenesaca, Mariana Bomfim Soares

**Affiliations:** 1 Department of Agricultural Production Sciences, São Paulo State University (UNESP), Jaboticabal, São Paulo 14884-900 Brazil; 2 Department of Plant Pathology, Federal University of Viçosa (UFV), Viçosa, Minas Gerais 36570-090 Brazil

**Keywords:** Plant development, Plant stress responses

## Abstract

Adopting a Si supply strategy can amplify the sugarcane response. Thus, this study aimed to verify whether Si supply in the pre-sprouted seedling (PSS) formation phase would have an effect after field transplanting similar to Si supply only in the field phase (via foliar spraying or fertigation). Furthermore, this study aimed to verify whether Si supply in the PSS formation phase associated with Si fertigation after transplanting can potentiate or amplify Si benefits. Two experiments were conducted. In experiment I, pre-sprouted seedlings were grown in a nursery without Si (Control) and with Si. Experiment II was conducted in the field on Eutrustox soil with the following treatments: no Si supply (Control); Si supplied during the PSS formation phase; Si supplied through foliar spraying in the field; Si supplied through fertigation in the field; Si supplied in the PSS formation phase and during field development. Silicon used in both crop phases benefited sugarcane by increasing photosynthetic pigment content and the antioxidative defense system. The innovation of Si management to be supplied via fertigation integrated with both crop phases (PSS and in the field) optimizes the element’s use by increasing the crop's productivity and sustainability.

## Introduction

Sugarcane (*Saccharum officinarum* L.) is an important renewable energy source, and there is a demand for its increased production worldwide^1–4^. Moreover, the increasing need to replace non-renewable energy from oil with bioenergy is part of international strategies to mitigate greenhouse gases affecting climate change.

The use of pre-sprouted seedlings (PSS) has expanded in the sugarcane planting stage. It comprises the development of a sugarcane plant from a cut part of the stem containing only one bud. It is an alternative to the conventional method, which uses stem seeds containing three to four buds^5,6^. Pre-sprouted seedlings’ use has advantages since they undergo rigorous phytosanitary treatment, and the genetic material is homogeneous, reflecting the crop's productive potential. However, since this system uses a mini stem, it has little water and nutrient reserves. These characteristics make the material very sensitive after transplanting due to the various stresses in the field.

Beneficial elements supply, such as silicon (Si), has been used to favor plant development. Silicon can favor crop growth, with or without stress^7,8^, due to its physiological benefits. It increases photosynthetic pigment biosynthesis, strengthens the antioxidant defense system, and favors photosynthetic efficiency, a fact observed in different species^9–12^, including sugarcane^13,14^. Furthermore, Si benefits are favored in sugarcane because the crop has a high root uptake efficiency regarding this element^15–17^.

Studies evaluating Si in sugarcane in the field predominantly use solid silicates, such as calcium silicate^18,19^, which has very low solubility in water and is used in high doses in the soil.

Recent studies have indicated the use of soluble sources, based on potassium silicate or sodium silicate, which allow a reduction in the element doses and maintain the biological benefits in the plants^8^. Because they have high soluble Si content, these sources can increase the initial growth of pot-grown PSS when supplied via foliar sprays^16,20^, or, more prominently, via fertigation^14,21^. However, these seedlings were not transplanted into the field. It is possible that using a PSS enriched with Si and transplanted to the field can favor the crop's best development, which can be potentiated if there is a complement of the element in the field (via foliar spraying or fertigation). There are indications that soluble Si, even without stress, favors the crop’s photosynthetic rate and initial growth^22^, and consequently, biomass production^23^. However, there needs to be more information to verify whether these beneficial effects of soluble Si on the initial growth of sugarcane grown in pots seen in previous studies are sustained long enough to increase stem growth and productivity at the field level.

In this context, an important challenge would be to advance research to define the best management and form of Si supply, employing soluble sources, to potentiate its absorption and physiological aspects to integrate the PSS production and field phases.

Therefore, the following hypotheses arise: (i) fertigation with Si in PSS production has immediate effects on their growth and residual effects by increasing crop productivity after transplanting to the field; (ii) using PSS fertilized with Si has a residual effect on crop productivity after transplanting similar to foliar spraying performed in the field; (iii) using PSS fertilized with Si associated with fertigation with the element after transplanting stands out in increasing crop productivity; (iv) Si beneficial mechanisms may be due to physiological improvements in sugarcane that reflect in crop productivity, occurring proportionally to the Si content increase in the plant promoted by this element's different application methods.

Thus, this study aimed to verify whether Si supply in the pre-sprouted seedling (PSS) formation phase would have an effect after field transplanting similar to Si supply only in the field phase (via foliar spraying or fertigation). Furthermore, this study aimed to verify whether Si supply in the PSS formation phase associated with Si fertigation after transplanting can potentiate or amplify Si benefits.

## Results

### Experiment I

**Figure 1:**
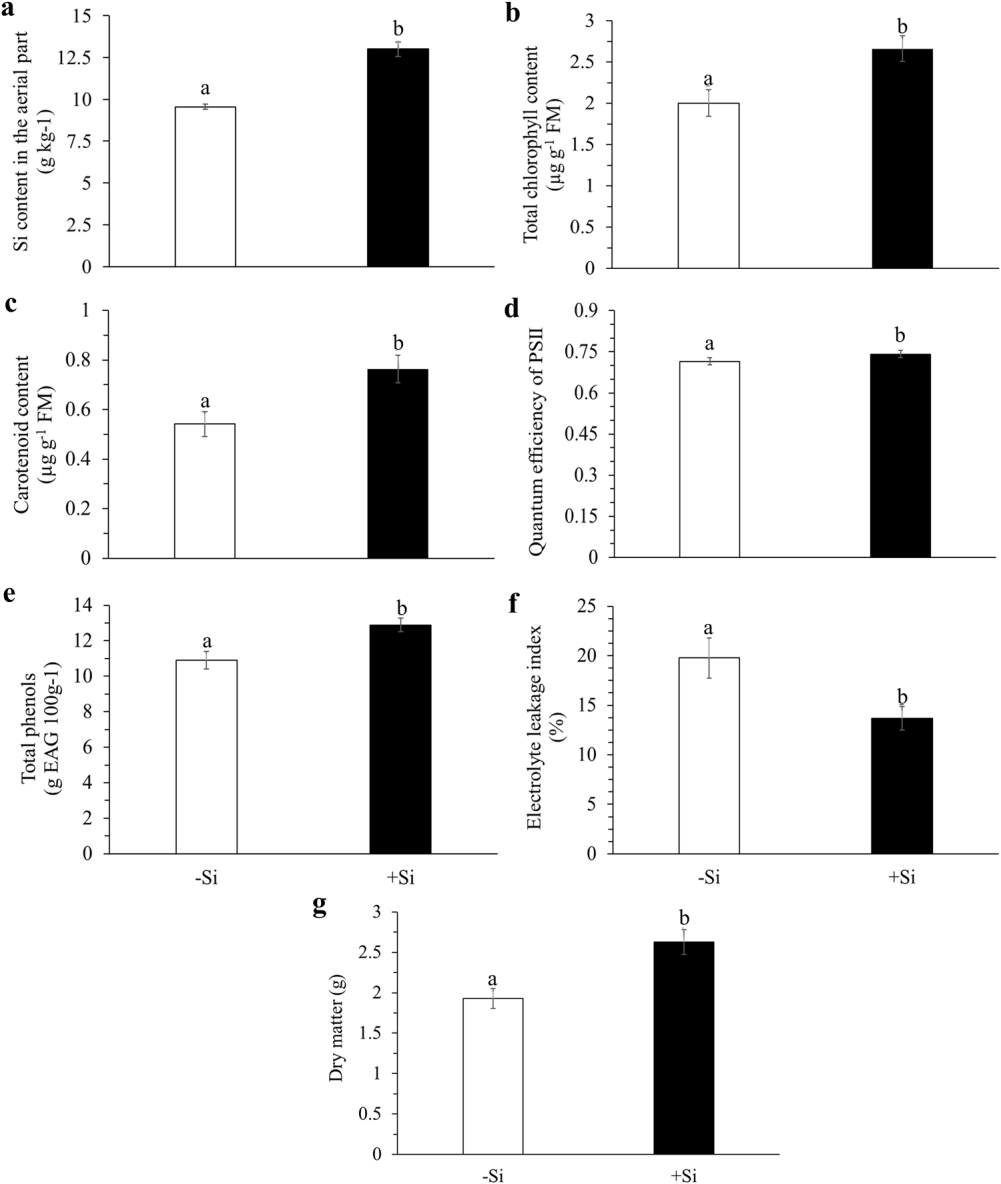
Silicon content in the aerial part (**a**), total chlorophyll content (**b**), carotenoid content (**c**), quantum efficiency of PSII (**d**), total phenol content (**e**), cellular electrolyte leakage index (**f**), and aerial dry matter production (**g**) in pre-sprouted sugarcane seedlings grown without (-Si) and with Si (+ Si) supply via fertigation. The error bars represent the standard error of the means. Lowercase letters differ between treatments according to the LSD test at 5% probability.

Silicon supply via fertigation provided a higher content of the element in the PSS (13.0 g kg^−1^) when compared to the control PSS (9.5 g kg^−1^), with a 36% increase in the element’s content (Fig. 1a). Moreover, Si fertigation increased the total chlorophyll (Fig. 1b) and carotenoid contents (Fig. 1c), the quantum efficiency in photosystem II (Fv/Fm) (Fig. 1d), and total phenols (Fig. 1e). In addition, it decreased the cellular electrolyte leakage (Fig. 1f) when compared with no Si supply (control). As a result, Si application increased the dry mass production of the PSS's aerial part (Fig. 1g). This increase was strongly correlated with the Si content in the aerial part (r = 0.72**) (Table 1).

**Table 1:** Pearson correlation (p ≤ 0.05) between the variables analyzed regarding experiments I (PSS phase) and II (field phase).

	Si content	Total chlorophyll	Carotenoid	Fv/Fm	Total phenol	CEL^1^	Height	Tillering	Elongation rate
**Experiment I**									
Total chlorophyll	0.41^ns^	–	–	–	–	–	–	–	–
Carotenoid	0.48*	0.86**	–	–	–	–	–	–	–
Fv/Fm	0.60**	0.38^ns^	0.37^ns^	–	–	–	–	–	–
Total phenol	0.60**	0.18^ns^	0.34^ns^	0.23^ns^	–	–	–	–	–
CEL	− 0.31^ns^	0.18^ns^	− 0.25^ns^	− 0.40^ns^	− 0.32^ns^	–	–	–	–
Dry mass	0.72**	0.43^ns^	0.60**	0.49*	0.56*	− 0.23^ns^	–	–	–
**Experiment II**									
Total chlorophyll	0.90**	–	–	–	–	–	–	–	–
Carotenoid	0.87**	0.95**	–	–	–	–	–	–	–
Total phenol	0.82*	0.87**	0.87**	–	–	–	–	–	–
CEL^1^	− 0.10^ns^	− 0.09^ns^	− 0.12^ns^	–	− 0.17^ns^	–	–	–	–
Height	0.95**	0.87**	0.86**	–	0.87**	− 0.11^ns^	–	–	–
Tillering	0.94**	0.90**	0.83**	–	0.84**	− 0.03^ns^	0.92**	–	–
Elongation rate	0.94**	0.89**	0.84**	–	0.86**	− 0.07^ns^	0.95**	0.96**	–
Productivity	0.94**	0.87**	0.82**	–	0.88**	− 0.07^ns^	0.93**	0.94**	0.93**

^ns^: p > 0.05. CEL^1^: cellular electrolyte leakage.

### Experiment II

**Figure 2:**
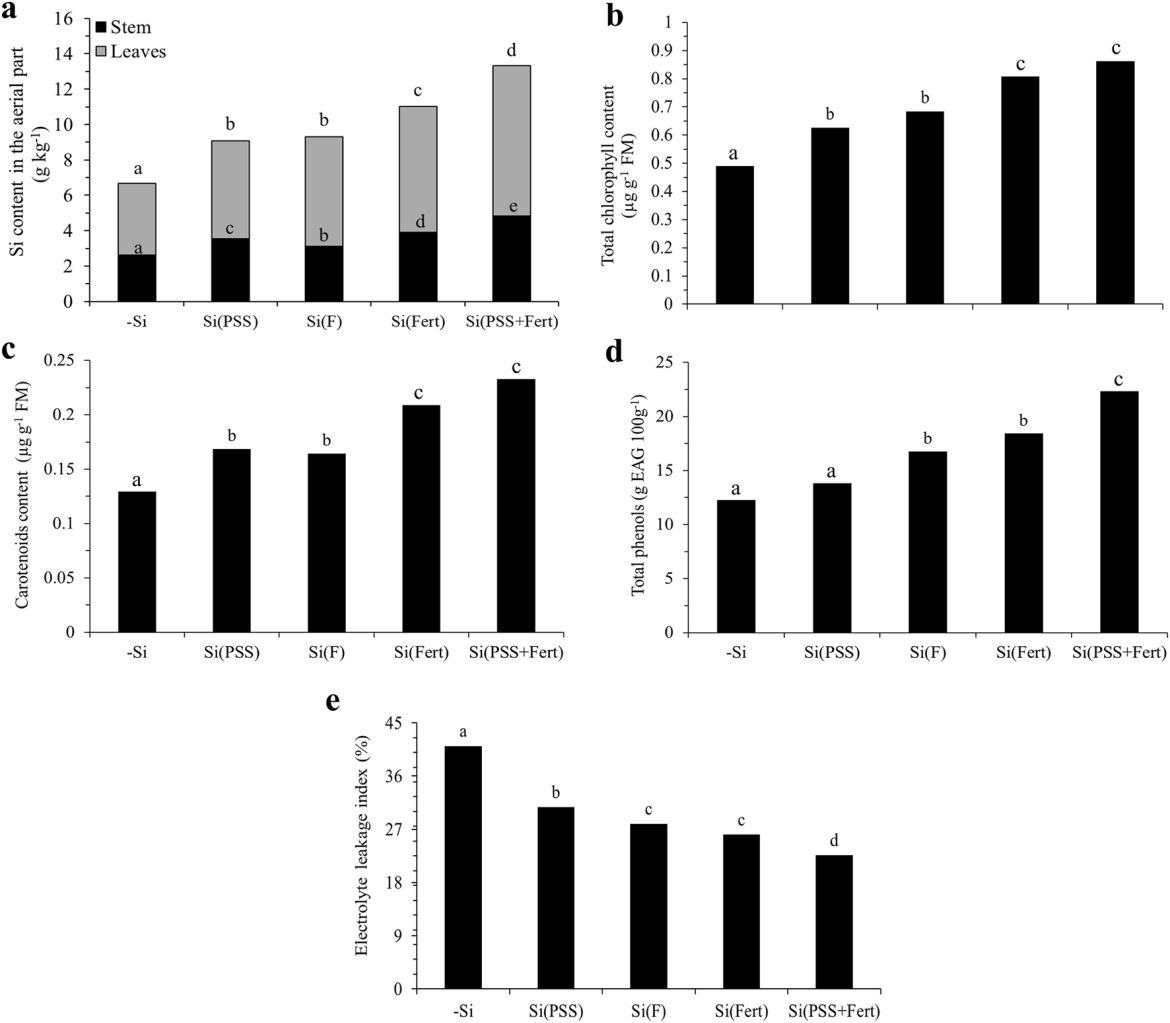
Silicon content in the aerial part of plants (**a**), total chlorophyll content (**b**), carotenoid content (**c**), total phenol content (**d**), and cellular electrolyte leakage index (**e**) in sugarcane grown without and with Si supply during different developmental stages: no Si supply (-Si); Si supply at the pre-sprouted seedling formation phase [Si(PSS)]; Si supply via foliar spraying [Si(F)]; Si supply via fertigation in the field [Si(Fert)]; and Si supply at the pre-sprouted seedling formation phase and in crop development [Si(PSS + Fert)]. Lowercase letters differ between treatments according to the LSD test at 5% probability.

Silicon supply only at the formation stage of the PSS (Si Seedling) or via foliar spraying (Si Foliar) efficiently increased Si content in the aerial part of sugarcane plants (9.08 and 9.32 g kg^−1^, respectively) when compared to the control treatment (6.67 g kg^−1^) (Fig. 2a). However, Si fertigation only in the field phase (Si Fert) provided higher Si content (11.0 g kg^−1^) in the aerial part of the plants compared to Si Seedling and Si Foliar. Silicon fertigation in the PSS production phase combined with field fertigation during plant development (Si Seedling + Fert) provided higher Si content (13.3 g kg^−1^) in the aerial part of sugarcane plants compared to the other treatments (Fig. 2a).

Sugarcane plants from the Si Seedling treatment and the Si Foliar treatment showed higher total chlorophyll content (0.626 and 0.684 µg g^-1^ FM, respectively) and higher carotenoid content (0.169 and 0.164 µg g^−1^ FM, respectively) when compared to plants from the control treatment (0.490 and 0.130, respectively). Furthermore, both Si Fert and Si Seedling + Fert treatments provided even greater increments in total chlorophyll (0.808 and 0.862 µg g^−1^ FM, respectively) (Fig. 2b) and carotenoids (0.209 and 0.233 µg g^−1^ FM, respectively) (Fig. 2c) when compared to the other treatments.

The Si Seedling + Fert treatment promoted a higher total phenol production (22.3 g EAG 100 g^−1^) (Fig. 2d) and a lower cellular electrolyte leakage (23%) (Fig. 2e) than the other treatments. Moreover, the Si Foliar and Si Fert treatments showed a higher total phenol production (16.76 and 18.41 g EAG 100 g^−1^) (Fig. 2d) and a lower cellular electrolyte leakage rate (28 and 26%) (Fig. 2e) than the Si Seedling and control treatments.

**Figure 3:**
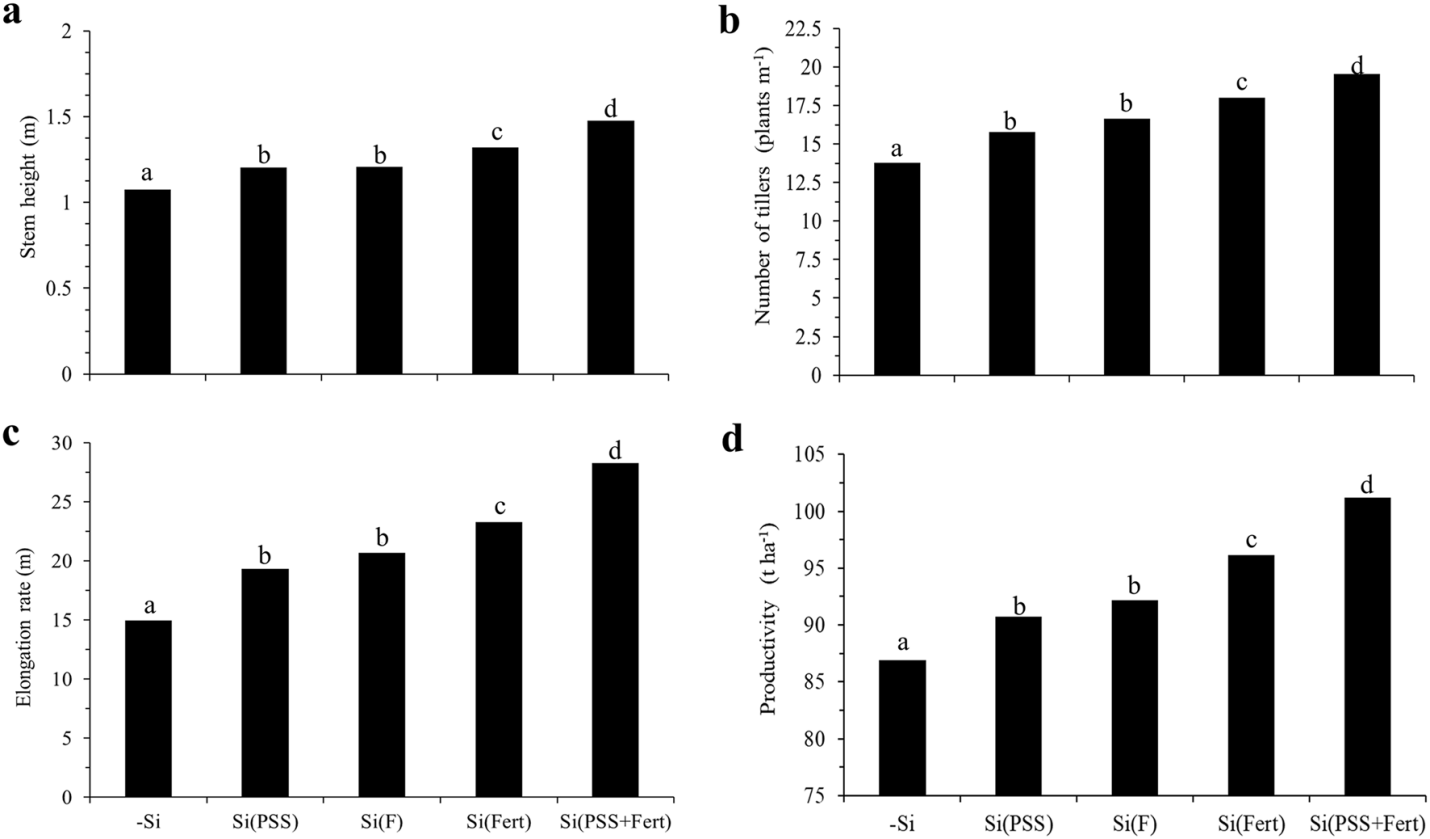
Stem height (**a**), number of tillers (**b**), elongation rate (**c**), and productivity (**d**) of sugarcane grown without and with Si supply during different developmental stages: no Si supply (-Si); Si supply at the pre-sprouted seedling formation phase [Si(PSS)]; Si supply via foliar spraying [Si(F)]; Si supply via fertigation in the field [Si(Fert)]; and Si supply at the pre-sprouted seedling formation phase and in crop development [Si(PSS + Fert)]. Lowercase letters differ between treatments according to the LSD test at 5% probability.

The Si Seedling and Si Foliar treatments showed similarities in the biometric evaluation, which reflected in the crop's productivity and reached 91 and 92 t ha^−1^, respectively (Fig. 3). However, the Si Fert treatment stood out from these treatments in these variables. Furthermore, the Si Seedling + Fert treatment stood out from the other treatments by presenting higher values for stem height (1.48 m) (Fig. 3a), tiller number (18.8 per linear meter) (Fig. 3b), stem elongation rate (1 m:28 m) (Fig. 3c), and productivity (101 t ha^−1^) (Fig. 3d). It should be noted that there was a strong correlation between the Si content in the aerial part of sugarcane plants and the biometric variables, especially stem height (r = 0.94**) and productivity (r = 0.93**) (Table 1).

## Discussion

The use of soluble Si sources in sugarcane crops is recent, initiating studies in PSS with promising results in the crop due to the increments in Si uptake when applied via foliar spraying or fertigation evaluated in stressed plants^13,20,21^. However, we also observed similar results in our studies with soluble Si with PSS but grown unstressed (Fig. 1a). It has been reported that unstressed plants have higher Si uptake than stressed plants^14^, possibly favoring biological gains for the plants.

Moreover, there have been doubts regarding the feasibility of soluble Si application via solution (fertigation or foliar spraying) to field-grown sugarcane in increasing the crop's Si uptake. The studies in this condition included Si sources (calcium silicate) with very low water solubility, besides the use of relatively high doses, which were incorporated into the soil in total area (up to 600 kg ha^−1^ of Si)^22^, or in planting furrows (55 up to 165 kg ha^−1^ of Si)^18,19^.

Our study showed for the first time that applying soluble Si at relatively low doses on sugarcane under field conditions, by foliar spraying in three sprays (50.4 g ha^−1^ of Si) or by fertigation in 15 applications (2.3 kg ha^−1^ of Si), effectively increased the element’s content in the crop (Fig. 2a) and may have favored the plant's uptake. We believe that the Si content increase in the plant from Si solutions applied via foliar spraying or fertigation is due to the diluted soluble Si form (≤ 3 mM of Si). It avoids polymerization processes that could decrease Si uptake by the plant^23^. In the soil solution, Si is in ionic form (SiO_3_^2−^ and SiO_4_^4−^)^24^, which is not polymerized at diluted concentrations. These results regarding the potential use of Si in sugarcane solutions have important practical implications for changing the Si application philosophy in the field. Furthermore, they can directly impact the cost/benefit ratio of silicate fertilization in the crop due to relatively low Si doses.

Another unprecedented finding was that using PSS that received Si during their formation in the nursery enriched PSS with Si (Fig. 1a), which was important to increase the Si content in the field crop after transplanting (Fig. 2a), indicating an accumulative Si effect. Thus, it has been confirmed that the Si in PSS can be transferred to the sugarcane crop in the field, an important fact that justifies using PSS enriched with Si. Furthermore, it may indicate that some of the Si in PSS can be mobilized to the sugarcane leaves, probably from the soluble Si in the plant, because most of the Si in the plant is polymerized (SiO_2_.nH_2_O) in the cell wall of the epidermis cells, remaining stationary in the plant^25^.

The Si accumulative effect found in our study is due to the high efficiency in the element’s uptake by sugarcane, especially because of the use of soluble Si, avoiding or reducing the element’s polymerization^23^. However, other effects are also important, such as the presence of sorbitol, which stabilizes Si and prevents the element's polymerization^26,27^, associated with the fact that the solution's acidification (pH (water): 5.5) increases the element's monomeric species (H_4_SiO_4_)^28,29^, favoring plant uptake. Furthermore, sugarcane belongs to the Poaceae family, which has specific proteins with a high affinity for Si uptake, classifying the crop as a Si accumulator^30^.

It is well-known that vital physiological processes in plants benefit from increased Si uptake^8^. This study evidenced the important role of Si supply in increasing the contents of total chlorophyll and carotenoids in the PSS leaves (Fig. 1b,c) (Experiment I) and the sugarcane leaves grown in the field (Fig. 2b,c) (Experiment II), emphasizing the plants in the Si Fert and Si Seedling + Fert treatments. Therefore, the increase in Si content in PSS or plants in the field demonstrated this element's role in structuring chloroplasts, allowing the increase in photosynthetic pigments^31,32^. Moreover, the Si supply allowed a decrease in energy losses in the form of fluorescence in PSS (Fig. 1d) (Experiment I) due to the increase in EQFII (Fv/Fm), which we can ascribe to the element's role in maintaining the photosynthetic apparatus integrity^33^.

Silicon also plays an important role in strengthening the plants' antioxidant defense system^34^. We evidenced that Si supply promoted an increase in total phenols in the PSS in the nursery phase (Experiment I) (Fig. 1e) and after field transplanting (Experiment II), especially in the Si Seedling + Fert treatment (Fig. 2d). Phenolic compounds are important antioxidants because they reduce lipid oxidation and mitigate plant damage^35^. In other words, they improve the antioxidant defense system. This Si benefit is evidenced by the fact that Si decreased the cellular electrolyte leakage in the seedlings (Experiment I) (Fig. 1f) and the field crop (Experiment II), especially in the Si Seedling + Fert treatment (Fig. 2e). The decrease in electrolyte leakage due to increased Si content in the plant occurs because the element favors cell membrane integrity^34,36^.

The Si effects on the nutritional and physiological aspects increased the PSS biomass (Fig. 1g) (Experiment I) and the biometric variables of the crop in the field (Fig. 3) (Experiment II), reflecting in the sugarcane productivity. The biometric variables’ behavior followed the increased Si content in the plants in the field, providing higher values for stem height (Fig. 3a), number of tillers (Fig. 3b), stem elongation rate (Fig. 3c), and crop productivity (Fig. 3d). The Si Seedling + Fert treatment stood out, followed by the Si Fert treatments. However, the Si Foliar treatment was similar to the Si Seedling. Thus, our data support the literature, which also showed increased sugarcane productivity due to increased stem height, number of tillers^37–39^, and Si content in the plant^18^.

Our results indicate that the Si supply in the PSS production phase combined with the element’s fertigation after the crop transplanting to the field comprises a new concept for integrated silicate fertilization in sugarcane crops. This method’s efficiency for plant nutrition doubled the Si content in the plant compared to the control treatment. Moreover, it favored the plant’s physiological and growth aspects, resulting in a 16% increase in crop productivity. Therefore, using relatively low Si doses (< 2.4 kg ha^−1^), efficiently employed with a soluble source, such as sodium silicate, reinforces the importance of the source’s quality for Si use in sugarcane crops. Furthermore, economically, silicates are relatively affordable, costing 80% to 90% less than other fertilizers^40^.

Notably, the PSS enriched with Si in the formation phase showed a response in the field similar to the sugarcane plants that received the element only via foliar spraying. It is possible to ascribe this behavior to the element’s benefits in increasing the Si content in the plant, which was enough to increase photosynthetic pigments (Fig. 1b; Fig. 2b). Therefore, Si-enriched PSS can replace the element’s application in the field, avoiding the need for several Si foliar sprays, which would decrease production costs and indicate high feasibility.

Thus, the results indicate that all of this study’s hypotheses can be accepted since we observed immediate and residual effects on the Si-enriched PSS (i), in addition to the PSS showing similar productivity when compared to the plants that received the element’s foliar spray (ii). Furthermore, we found that using PSS enriched with Si associated with Si fertigation in the field increases crop productivity (iii). We can ascribe this fact to the higher Si content in the plant, reflecting physiological improvements and sugarcane productivity (iv). It was reinforced by the strong correlation between Si content in plants and physiological aspects and the sugarcane growth and productivity variables.

This study’s results reinforce that Si may have benefitted sugarcane, a fact also reported by other authors, but in the early growth stage^41^ and other species^42–44^. Therefore, although many more studies evaluate the Si benefits in stressed plants, it is possible that this also occurs in unstressed plants^7^. The evidence shows that Si should be included among the elements that significantly influence plant life^45^. Thus, our discovery opens the path for this element's wide use in sugarcane crops, strengthening their sustainability.

As such, the study proposes a new system that changes the Si-fertilization concept in sugarcane based on an integrated program. It starts with Si-enrichment in the PSS formation and their transplanting to the field, complemented by the crop’s Si fertigation, which should directly impact the crop’s sustainability. The future perspective is that further research can advance studying the Si fertigation effects in the field with systematic approaches to integrate PSS nutrition with the crop in the field, studying different soils and sugarcane cultivars.

## Future expectations

If this study’s hypotheses are accepted, it will open for the first time new perspectives for using a silicate fertilization program integrating the PSS production phase and the field phase. In other words, even without stress, stem production favors sustainable cropping, benefiting different regions of the world that cultivate this species and use PSS.

## Methods

### Study location

Two experiments were conducted at Fazenda Palmital in Jaboticabal, São Paulo, Brazil (21°10′24.762″S 48°17′19.838″W). According to the Köppen climate classification, the region's climate is humid tropical (Aw). Furthermore, the microscale in the experiment region confirms the climate. That is, it is humid tropical with dry winter^46^.

### Experiment I

The first experiment was conducted with PSS sugarcane cultivar CTC 9001, acquired from a commercial nursery, having four leaves emerging from the bud. The sugarcane variety CTC 9001 was developed by the Sugarcane Technology Center. All plant studies followed relevant institutional, national, or international guidelines and regulations. Our research did not include endangered species and followed the Declaration of IUCN Policy on Research Involving Endangered Species. During the PSS development, the average minimum temperature was 16.2 °C and the maximum 31.7 °C.

Pre-sprouted seedlings were submitted to the following treatments: without Si supply (control) and with Si supply via fertigation, with 15 repetitions, arranged in an entirely randomized design. The experimental unit comprised a plastic tray having cells (140 cm^3^) filled with vermiculite containing a PSS.

Silicon fertigation was applied at 5 cm above the PSS, starting at the formation process, employing a Si concentration of 2 mmol L^−1^ in the form of sodium silicate (188.6 g L^−1^ of Si and 50.1 g L^−1^ of Na) at 2, 6, 10, 14, 18, 22, 26, and 30 days after the beginning of the formation phase, totaling eight applications. The definition of the Si concentration for fertigation followed the value indication used in several studies with different crops^47^. Moreover, the PSS received a supply of nitrogen (N), phosphorus (P), and calcium (Ca) every ten days, at 0.05, 0.12, and 0.04 g per PSS, respectively, through a 15 mL solution per cell. The sources used included monoammonium phosphate and calcium nitrate.

In order to irrigate the PSS, the reference evapotranspiration was considered, and 80% of the previous day’s value was replaced, based on data from the Unesp Agroclimatological Station, Jaboticabal Campus.

At 30 days after the beginning of applying the treatments, evaluations were performed on the + 1 leaf (first leaf from top to bottom, with fully visible sheath) of the PSSs, which are described below.

### Photosynthetic pigments

The total chlorophyll and carotenoid content were determined by depigmentation using acetone (80%) and reading in a photometer spectrum^48^. Two 6 mg discs were collected in the middle third of the leaf limb. Readings were taken in a spectrophotometer at 663 nm (chlorophyll a) and 470 nm (carotenoids), and the content was defined based on fresh matter.

### Quantum yield of PSII (Fv/Fm)

The quantum efficiency of the PSII (variable fluorescence and maximum fluorescence ratio) of leaves was measured using a fluorometer (Opti-Sciences® – Os30p +).

### Phenolic compounds content

The analysis of the phenolic compounds content followed the colorimetric reaction method and a spectrophotometer reading^49^.

### Cellular electrolyte leakage index

Collections of five leaf discs with a 5 mm diameter were made to analyze the electrolyte leakage index by heating in an autoclave and reading using an electric conductivity meter^50^.

### Aerial part dry mass

Five PSS were collected per plot to obtain dry mass. First, the PSSs were washed with water, detergent solution (Extran® 0.1% v/v), acid solution (HCl 0.3% v/v), and deionized water. Then, they were dried in a forced air circulation oven at 65 ± 5 °C until reaching constant mass. Finally, the samples were weighed to determine the dry mass of the aerial part and ground in a Wiley-type mill.

### Silicon content

The Si content was determined using alkaline digestion with NaOH and carbon oxidation with H_2_O_2_, followed by reading in a colorimetric spectrophotometer^51^.

### Statistical analysis

The data were verified for normality (Shapiro–Wilk test) and homogeneity of variances (Levene test). They were subjected to analysis of variance by F-test (p ≤ 0.05), and the mean values of the treatments were compared using the LSD test (p ≤ 0.05). Pearson’s correlation coefficient was determined to measure the correlation between the study variables (p ≤ 0.05). Statistical analyses were performed using SAS® statistical software (Cary, NC, USA).

### Experiment II

**Figure 4:**
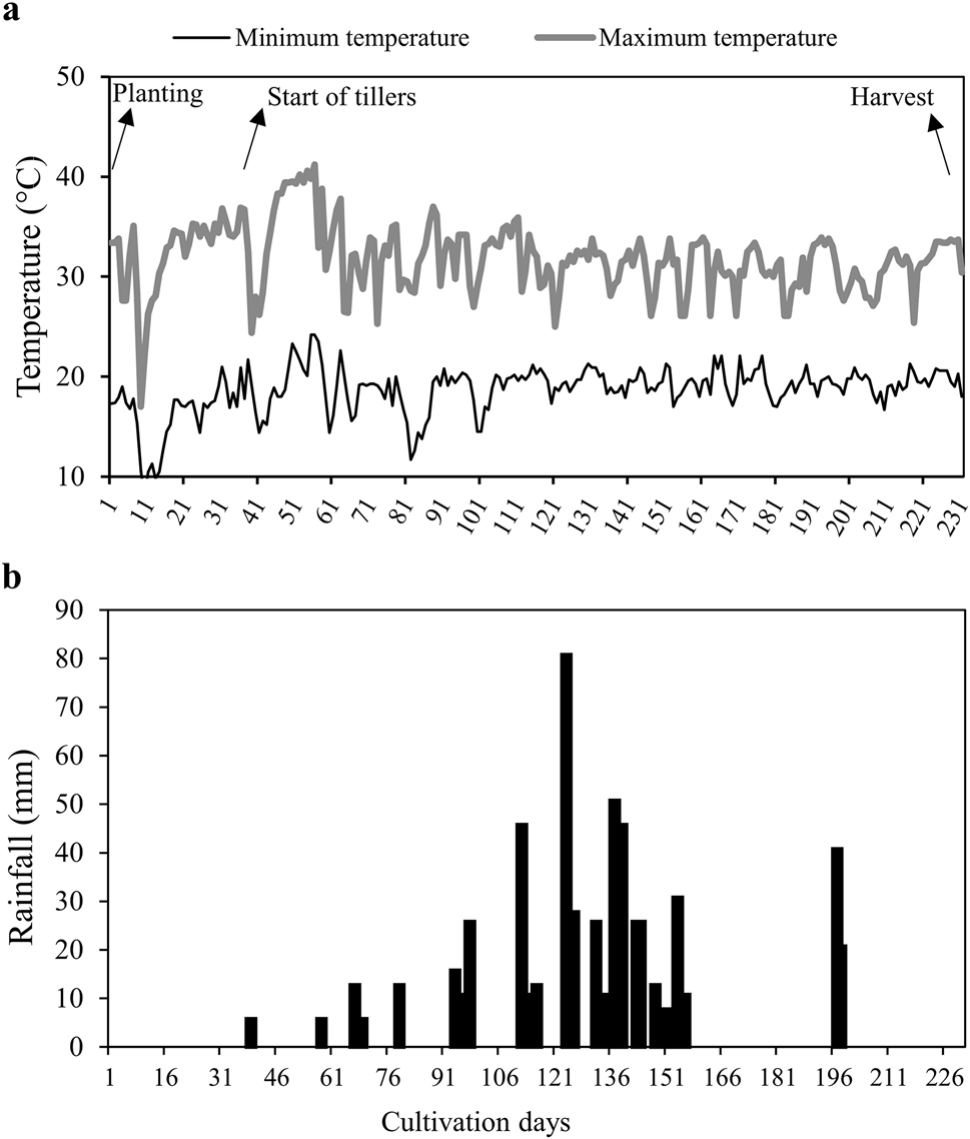
Maximum and minimum air temperature (**a**) and rainfall (**b**) during the development of experiment II.

The study was conducted in the field with the sugarcane variety CTC 9001 developed by the Sugarcane Technology Center. Air temperatures (°C) and rainfall (mm) data were recorded during the experimental period (Fig. 4).

The soil of the experimental area was classified as Eutrustox. Soil samples from the 0–0.2 m layer were collected to perform chemical analysis for fertility^52^ purposes and showed the following results: pH (CaCl_2_): 5.2; P (resin): 29.0 mg dm^−3^; organic matter: 23 g dm^−3^; K: 1.0 mmol_c_ dm^−3^; Ca: 40 mmol_c_ dm^−3^; Mg: 13.0 mmol_c_ dm^−3^; H + Al: 39.0 mmol_c_ dm^−3^; Al: 0.0 cmol_c_ dm^−3^. Furthermore, the soil’s available Si content (CaCl_2_ extractant) (10 mg dm^−3^) was determined according to the colorimetric method^49^ and was considered low content^53^.

Based on the soil's chemical analysis, the lime requirement was calculated to increase the base saturation (V) (V = (sum of Ca + Mg + K content/cation exchange capacity × 100)) to 70%. Therefore, lime was applied to the soil surface and incorporated into the soil in the 0–20 cm depth layer.

Subsoiling and harrowing operations were performed in the area. First, planting furrows 0.35 m deep were made. Then, an organic compost (4 t ha^−1^ chicken litter and 10 t ha^−1^ filter cake) and fertilizers containing 7 kg ha^−1^ of N, 34 kg ha^−1^ of P_2_O_5_, and 11 kg ha^−1^ of K_2_O were applied to employ the ammonium sulfate, triple superphosphate, and potassium chloride sources.

The herbicide metolachlor (2 L ha^−1^) was used, classified as pre-emergent, and applied 15 days before the sugarcane planting (PSS) to control weeds. During the mother lines cultivation, 40 kg ha^−1^ of N and 15 kg ha^−1^ of K_2_O were applied at 70 days after planting.

After planting the PSS, the plants were irrigated with a daily rate of 30 mm for six consecutive days to ensure adequate seedling establishment in the field.

The variety was submitted to the following treatments: no Si supply (Control); Si supply during the formation period (Si Seedling); Si foliar spraying (Si Foliar); Si supply via fertigation after planting (Si Fert); and a combination of Si Seedling and Si Fert (Si Seedling + Fert), with five repetitions, arranged in randomized blocks. The plots comprised of a mother line, which was 7.2 m long.

The Si supply in the PSS of the Si Seedling treatment started during the formation process, as reported in Experiment I. Fertigation with Si in the field treatments Si Root and Si RM (1.8 mmol L^−1^ of Si) were performed in 15 applications. Unlike the PPS used in the Si Root treatment, the PPS used in the Si RM treatment received Si in the formation phase before being transplanted to the field. Therefore, in the initial six-day period after transplanting the PSS to the field, daily irrigation (30 mm) was performed, followed by Si application. Then, nine applications of the element were made at 99, 113, 125, 127, 133, 138, 140, 145, and 155 days after transplanting the PSS right after rainfall (> 20 mm). In all 15 fertigation applications, the solution containing Si was applied directly to the planting line, with an amount equivalent to a 1.5 mm water slide.

The application of the Si Foliar treatment began in the stem formation phase four months after planting, when the plant presented a significant leaf area. Three applications were made at 15-day intervals. Silicon Foliar applications (3 mmol L^−1^) were carried out with relative humidity ≥ 60% using the Jactor 2030 uniport sprayer. The pH value of the grout was set to 5.5 ± 0.1 from phosphoric acid using Startec agricultural adjuvant (1 mL/100 L water) with a flow rate set to 200 L ha^−1^ of grout. Notably, the foliar application of Si did not reach the soil due to the overlapping of the sugarcane leaves at the time of spraying. Determining the Si concentration in the Si Foliar treatment followed literature recommendations^21^. In both treatments, the Si source comprised sorbitol-stabilized sodium silicate (169.7 g L^−1^ of Si and 50.1 g L^−1^ of Na).

One day before dismantling the experiment, 10 + 1 leaves were collected per plot. The collection was performed at 6 a.m., and the leaves were stored in a Styrofoam box with ice to preserve the material. Then, they were transported to the laboratory for the physiological analyses, as described in Experiment I.

The experiment was completed six months after planting, and biometric evaluations were performed on the plants in the 5.2 linear meters of the plot's central part. All the variables analyzed are described below.

### Height, number of tillers, elongation rate, and productivity

The stem height was obtained six months after planting using a tape measure, and the number of tillers in the 5.2 linear meters of the plot’s central part was counted and calculated to be expressed by the number of tillers in 1 m. Then, the elongation rate was calculated based on the metric sum of the stem heights in one linear meter of the sugarcane planting line. In other words, it expresses the multiplication level of each sugarcane linear meter into new stems to be used as seed-stem. Finally, all the stems in the 5.2 linear meters of the plot's central part were collected and weighed, and the productivity for one hectare was estimated.

### Silicon content in sugarcane stalks

The methodology used in Experiment I was followed to determine the Si content. Ten stalks were collected per plot, collected at random. First, the stalks were ground. Then, samples were collected, placed in paper bags, and taken for drying in a forced air circulation oven at 65 ± 5 °C. Finally, the samples were ground in a Wiley-type mill when they reached constant mass.

### Statistical analysis

The data were verified for normality (Shapiro–Wilk test) and homogeneity of variances (Levene test). They were subjected to analysis of variance by *F*-test (p ≤ 0.05), and the mean values of the treatments were compared using the LSD test (p ≤ 0.05). Pearson’s correlation coefficient was determined to measure the correlation between the study variables (p ≤ 0.05). Statistical analyses were performed using SAS® statistical software (Cary, NC, USA).

### Statement of handling of plants

The authors confirm that the handling of the plants followed the Declaration of IUCN Policy on Research Involving Endangered Species and the Convention on Trade in Endangered Species of Wild Fauna and Flora.

## Data Availability

This manuscript includes all data generated or analyzed during this study.
